# Development and characterization of a *Nannochloropsis* mutant with simultaneously enhanced growth and lipid production

**DOI:** 10.1186/s13068-020-01681-4

**Published:** 2020-03-05

**Authors:** Ae Jin Ryu, Nam Kyu Kang, Seungjib Jeon, Dong Hoon Hur, Eun Mi Lee, Do Yup Lee, Byeong-ryool Jeong, Yong Keun Chang, Ki Jun Jeong

**Affiliations:** 1 Department of Chemical and Biomolecular Engineering, Korea Advanced Institute of Science and Technology (KAIST), 291 Daehak-ro, Yuseong-gu, Daejeon, 34141 Republic of Korea; 2 Advanced Biomass R&D Center (ABC), KAIST, 291 Daehak-ro, Yuseong-gu, Daejeon, 34141 Republic of Korea; 3 Department of Agricultural Biotechnology, Seoul National University, Seoul, 08826 Republic of Korea; 4 Institute for the BioCentury, KAIST, 291 Daehak-ro, Yuseong-gu, Daejeon, 34141 Republic of Korea; 5 Present Address: Carl. R. Woese Institute for Genomic Biology, University of Illinois at Urbana-Champaign, Urbana, IL USA; 6 Present Address: School of Energy and Chemical Engineering, Ulsan National Institute of Science and Technology (UNIST), Ulsan, 44919 Korea; 7 Present Address: Single-Cell Center, Qingdao Institute of BioEnergy and Bioprocess Technology (QIBEBT), Qingdao, 266101 Shandong China

**Keywords:** Microalgae, *Nannochloropsis salina*, FACS, Insertional mutagenesis, Trehalose-6-phosphate synthase, Trehalose

## Abstract

**Background:**

The necessity to develop high lipid-producing microalgae is emphasized for the commercialization of microalgal biomass, which is environmentally friendly and sustainable. *Nannochloropsis* are one of the best industrial microalgae and have been widely studied for their lipids, including high-value polyunsaturated fatty acids (PUFAs). Many reports on the genetic and biological engineering of *Nannochloropsis* to improve their growth and lipid contents have been published.

**Results:**

We performed insertional mutagenesis in *Nannochloropsis salina*, and screened mutants with high lipid contents using fluorescence-activated cell sorting (FACS). We isolated a mutant, Mut68, which showed improved growth and a concomitant increase in lipid contents. Mut68 exhibited 53% faster growth rate and 34% higher fatty acid methyl ester (FAME) contents after incubation for 8 days, resulting in a 75% increase in FAME productivity compared to that in the wild type (WT). By sequencing the whole genome, we identified the disrupted gene in Mut68 that encoded trehalose-6-phosphate (T6P) synthase (TPS). TPS is composed of two domains: TPS domain and T6P phosphatase (TPP) domain, which catalyze the initial formation of T6P and dephosphorylation to trehalose, respectively. Mut68 was disrupted at the TPP domain in the C-terminal half, which was confirmed by metabolic analyses revealing a great reduction in the trehalose content in Mut68. Consistent with the unaffected N-terminal TPS domain, Mut68 showed moderate increase in T6P that is known for regulation of sugar metabolism, growth, and lipid biosynthesis. Interestingly, the metabolic analyses also revealed a significant increase in stress-related amino acids, including proline and glutamine, which may further contribute to the Mut68 phenotypes.

**Conclusion:**

We have successfully isolated an insertional mutant showing improved growth and lipid production. Moreover, we identified the disrupted gene encoding TPS. Consistent with the disrupted TPP domain, metabolic analyses revealed a moderate increase in T6P and greatly reduced trehalose. Herein, we provide an excellent proof of concept that the selection of insertional mutations via FACS can be employed for the isolation of mutants with improved growth and lipid production. In addition, trehalose and genes encoding TPS will provide novel targets for chemical and genetic engineering, in other microalgae and organisms as well as *Nannochloropsis*.

## Background

Recently, the importance of biofuels from biomass, which are sustainable and environmentally friendly alternative energy sources, are being highlighted in accordance with the introduction of the Renewable Fuel Standard (RFS) and increase in the planned biofuel supplement. The purposed volume of total advanced biofuels was proposed to be up to 5.04 billion gallons in 2020, which is a fivefold increase in the final volumes 10 years ago [1, 2]. Microalgae serve as feedstocks for biomass and biofuels because of their higher biomass productivity and photosynthetic efficiency compared to those of plants [3], and can be cell factories for the production of secondary metabolites, food additives, cosmetics, pharmaceuticals, and industrial enzymes [4]. Among the microalgal species, *Nannochloropsis* are considered a promising microalgae in industrial fields owing to their robust growth and high lipid contents (up to 60–70% of the dry weight), including polyunsaturated fatty acids (PUFAs) such as eicosapentaenoic acid (EPA) [5].

Despite these advantages, microalgae-based biofuels have drawbacks in terms of commercialization because of their high production cost. In this respect, the genetic and metabolic engineering of microalgae are being actively pursued to improve lipid production, which can contribute to reduced biofuel prices [6–9]. Strain development has focused on the overexpression of metabolic enzymes related to lipid biosynthesis [10–14] and transcriptional regulators [15–18], or the targeted mutagenesis of competitive pathways of lipid biosynthesis using TALEN, RNAi, and CRISPR/Cas9 techniques [19–22].

Although genomic and metabolic resources are available for providing genetic targets to improve lipid and biomass production in microalgae [23], it is necessary to identify novel metabolic and/or regulatory genes for further improvements [24]. This can be achieved by employing random mutagenesis and high-throughput screening techniques to identify genes that can contribute to improved lipid accumulation, mainly in the model microalgae *Chlamydomonas reinhardtii* [25–27]. Such random mutagenesis can be achieved through irradiation by UV [28, 29], *γ*, and X-rays [30], as well as heavy ion beams [31], or by chemical mutagens, including ethyl methanesulfonate (EMS) [29, 32], *N*-methyl-N′-nitro-N-nitrosoguanidine (MNNG) [33], and *N*-methyl-N-nitrosourea [34]. However, such random mutagenic techniques present difficulties in identification of the responsible gene(s); this can be solved by insertional mutagenesis [35, 36]. Insertional mutagenesis allows the identification of disrupted genes by molecular techniques that provide sequence information at the integration junction between the mutagenic plasmid and the neighboring genomic sequences using polymerase chain reaction (PCR) or whole genome sequencing [37, 38]. Specifically, a few PCR techniques have been developed, including TAIL PCR and its variant RESDA PCR [39]. These techniques, which have been successful in identifying integration sites in plants and algae, involve nested primers in the mutagenic plasmids and random primers targeted for the neighboring genomic sequences [16].

Fluorescence-activated cell sorting (FACS) is a state-of-the-art technique that allows high-throughput analyses of numerous cells followed by the isolation of cells fulfilling the selection criteria. In general, cell sorting using FACS is enabled by preset fluorescence signals followed by sorting of cells for studying physiological and cellular properties, protein engineering, and overproducing target molecules [40]. FACS can be employed to screen and isolate cells from the EMS [26] and insertional mutagenesis libraries [27]. Such mutagenic screens facilitate successful isolation of mutants that accumulate lipids at a threefold higher rate than that in the wild type (WT) [29]. FACS-based screening was also used for laboratory adaptive evolution in *Chlamydomonas* to improve lipid metabolism [25]. It has also been used for the isolation of *Nannochloropsis* strains from the coastal waters of Singapore, resulting in cells with a twofold increase in lipid accumulation [41].

**Fig. 1:**
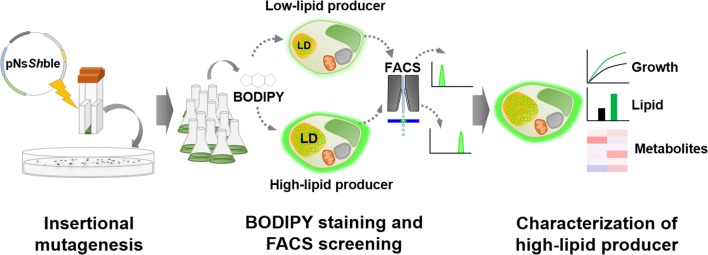
Schematic illustration of this study. Insertional mutagenesis and FACS-based screening by using BODIPY staining method were performed to isolated high-lipid biosynthesizing *N. salina*. And then, high lipid-producing *N. salina* mutant was characterized to seek novel findings which related to lipid biosynthesis in *N. salina*

In this study, we sought to identify novel genes associated with growth and lipid accumulation in *Nannochloropsis salina* by constructing an insertional mutant library and screening mutants with high lipid contents (Fig. 1). Based on FACS, one of the mutants, Mut68, showed the highest lipid contents and was thus, further characterized for growth, lipids, and other metabolites. Surprisingly, Mut68 showed both improved growth and lipid contents. Mut68 contained a single integration of the mutagenic plasmid, and we determined the disrupted gene by whole genome sequencing. Moreover, we found that the mutation occurred in a gene encoding TPS and determined consistent changes in metabolites, including trehalose and T6P. This study provides a novel target chemical and gene, i.e., trehalose and TPS, for chemical and genetic engineering to improve the production of biomass and lipids in *Nannochloropsis*, which can be further applied to other organisms.

## Results

### Isolation of insertional mutants for high lipid contents in *N. salina*

**Fig. 2:**
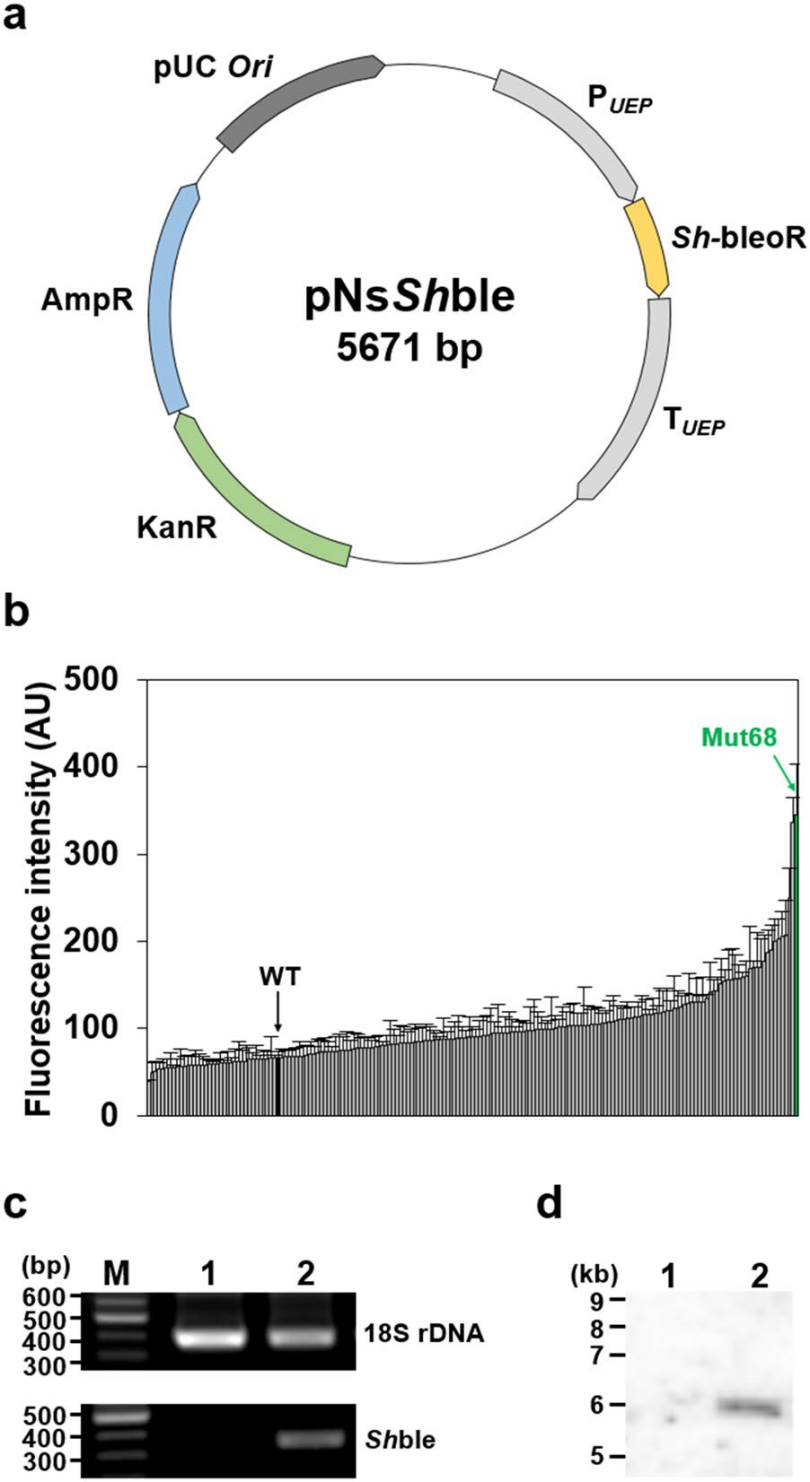
Insertional mutagenesis and FACS-based screening to isolate high lipid-accumulating *N. salina*. **a** Schematic map of pNs*Sh*ble. **b** FACS-based analysis for screening of insertional mutagenesis library. Microalgal cells were stained with BODIPY to detect the intracellular lipid in vivo. The stained samples were analyzed by using FACS and each of the mutants was arranged in the order of relative fluorescence intensity emitted from BODIPY. The X-axis represents the total analyzed single mutants, and the Y-axis represents the fluorescence detected from each mutants. The relative fluorescence intensity was calculated using mean value of four independent experiments. WT (black) and Mut68 (green) were indicated. **c** Confirmation of gene integration of Mut68 by performing PCR. M, marker; lane 1, WT; lane 2, Mut68. **d** Analysis of inserted copy number of pNs*Sh*ble by performing Southern blot analysis. Lane 1, WT; lane 2, Mut68

Insertional mutagenesis was performed to identify the genes associated with lipid metabolism in *N. salina*. The mutagenic plasmid, pNs*Sh*ble, which originated from *Streptoalloteichus hindustanus* (*Sh*-bleoR, UniProtKB: P17943) (Fig. 2a), contained a gene conferring bleomycin resistance for selection purposes. pNs*Sh*ble was introduced into the cells by electroporation, and 181 mutants were isolated. They were cultured for 7 days, stained with BODIPY for vital staining of lipids [42, 43], and subjected to FACS for the isolation of mutants with high lipid contents (Fig. 2b). We isolated a mutant that exhibited the highest lipid contents and named it Mut68.

### Molecular analysis of Mut68

Molecular analysis verified that only one gene was disrupted in Mut68 by the integration of the pNs*Sh*ble plasmid. To verify the presence of the insertion of pNs*Sh*ble, PCR was performed using F_*Sh*ble and R_*Sh*ble oligonucleotides to amplify the PCR product of the *Sh*-bleoR gene from the genomic DNA of the Mut68 (Fig. 2c; Additional file 1: Table S1). As a positive control for the presence of genomic DNA, amplification of the 18 s rDNA gene was also conducted using SR6 and SR9 oligonucleotides, and the 18S rDNA PCR product was observed in all lanes. In addition, Southern blot analysis was conducted to verify the copy number of the integrated pNs*Sh*ble fragment using a probe that binds to the *Sh*-bleoR gene; only one band for *Sh*-bleoR was detected in Mut68 (Fig. 2d, together with the whole blot in Additional file 2: Fig. S1). These results confirmed the presence of the mutagenic plasmid, and its integration at a single locus in the genome.

### Characterization of growth and lipid biosynthesis of Mut68

**Fig. 3:**
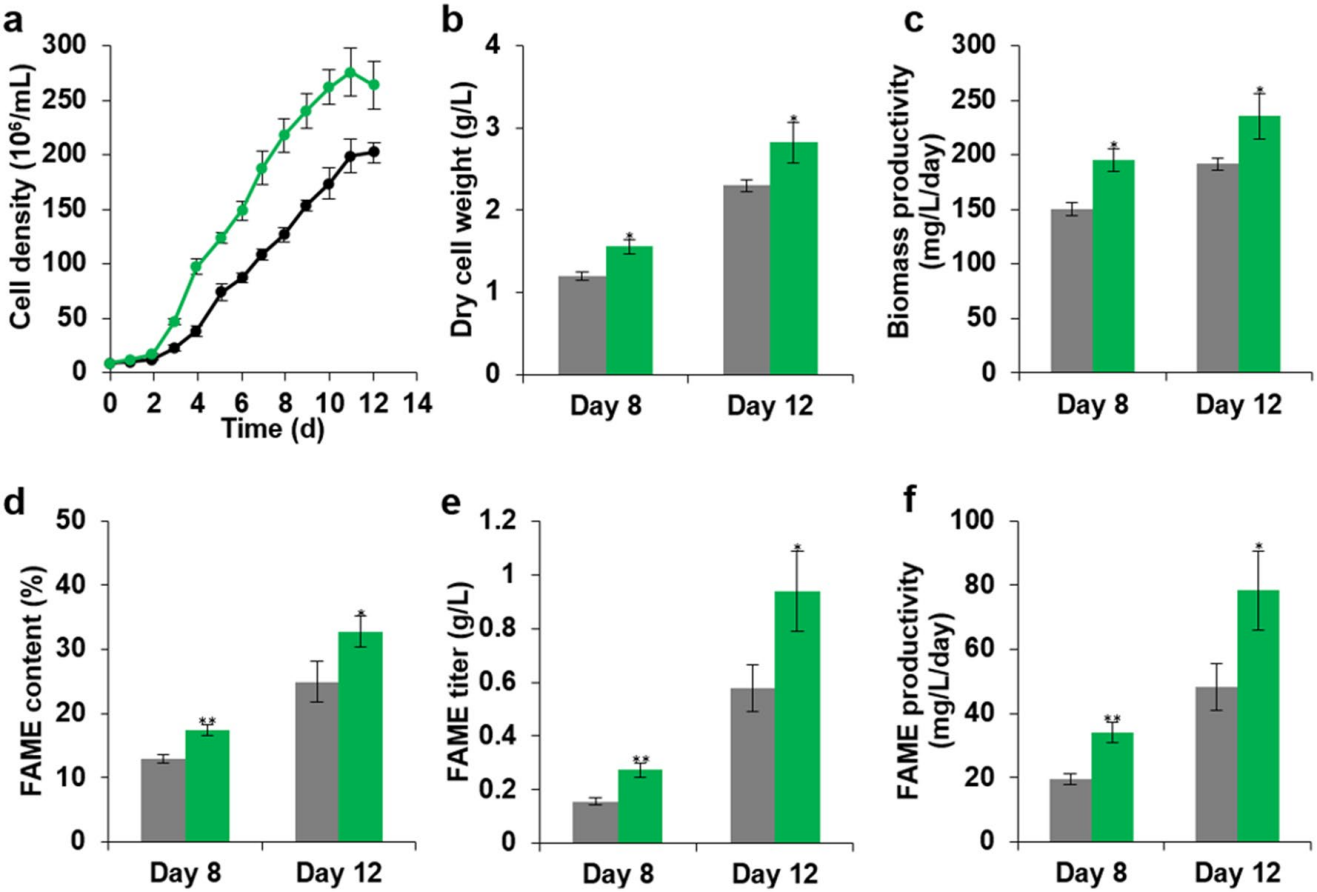
Comparison of growth and FAME profile of Mut68. **a** Growth curve, **b** dry cell weight, **c** biomass productivity, **d** FAME content, **e** FAME titer, **f** FAME productivity. Black, WT *N. salina*; green, Mut68. The data points represent the mean value of 4 samples, and error bar indicated by asterisks (* *p* < 0.05, ** *p* < 0.01, *** *p* < 0.001)

We characterized more phenotypes of Mut68, including the growth and fatty acid methyl ester (FAME) contents. As shown in Fig. 3a and Table 1, Mut68 showed a faster growth rate by 53%. The dry cell weight and biomass productivity of Mut68 were increased by 30% on day 8, and 23% on day 12 (Fig. 3b, c; Additional file 3: Table S2). The FAME content of Mut68 was increased by 34% on day 8 and 32% on day 12 compared to those of WT (Fig. 3d; Table 1). The FAME titer was also increased by 69% on day 8 and 62% on day 12 compared to those of the WT (Fig. 3e; Table 1). In addition, Mut68 exhibited a remarkable increase in FAME productivity by 75% on day 8 and 62% on day 12 (Fig. 3f; Table 1). In addition, the compositions of palmitic acid (C16:0) and palmitoleic acid (C16:1) were slightly increased in Mut68 (Additional file 4: Table S3). These results indicated that Mut68 accumulated more lipids without sacrificing growth or biomass, which are considered as ideal for the production of lipids for biofuel production.

**Table 1:** Specific growth rate and FAME analysis of WT *N. salina* and Mut68

Strain	Specific growth rate (day^−1^)	FAME content (%)		FAME titer (g/L)		FAME productivity (mg/L/day)	
		Day 8	Day 12	Day 8	Day 12	Day 8	Day 12
WT	0.57 ± 0.12	13.0 ± 1.4	24.9 ± 6.3	0.16 ± 0.03	0.58 ± 0.17	19.5 ± 3.5	48.3 ± 14.4
Mut68	0.87 ± 0.10*	17.4 ± 1.7**	32.8 ± 4.7*	0.27 ± 0.05**	0.94 ± 0.30*	34.1 ± 6.6**	78.3 ± 24.8*

The data show the mean value of 4 samples. As determined by Student’s *t*-test, significant differences are indicated by asterisks (* *p* < 0.05, ** *p* < 0.01, *** *p* < 0.001). Specific growth rate was calculated as (μ/day) = 1n (*X*_2_ – *X*_1_)/(*t*_2_ – *t*_1_), *X*_1_ and *X*_2_ are the initial and final biomass and *t*_1_ and *t*_2_ are the initial and final culture times

### Identification of the gene disrupted in Mut68

**Fig. 4:**
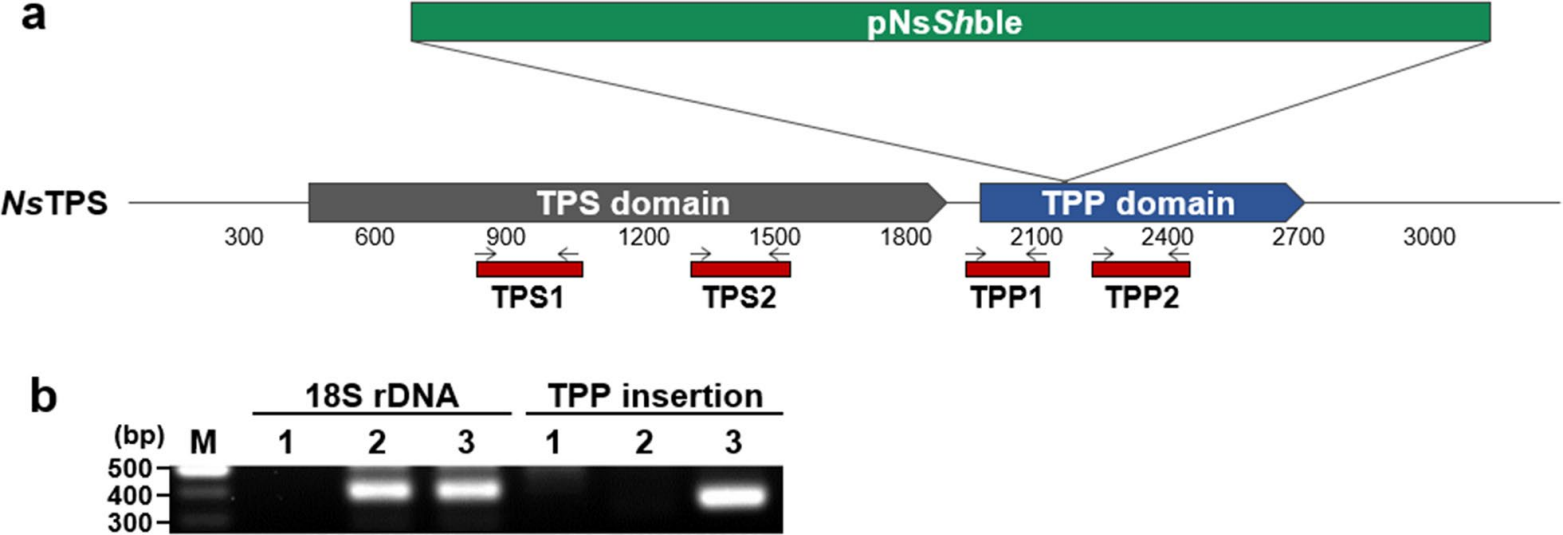
Genetic map of *Ns*TPS with integration site of pNs*Sh*ble. **a** Genetic map of *Ns*TPS is shown in approximate scale showing the TPS and TPP domains, together with integration site of pNs*Sh*ble. Locations of primers used for genomic PCR and qRT-PCR are shown. Amplicons of TPS1 and TPS2 were located in the TPS domain, while TPP1 and TPP2 were in the TPP domain. **b** Genomic DNA PCR was performed to confirm the integration site revealed by whole genome sequencing. M, marker; lane 1, pNs*Sh*ble control; lane 2, WT; lane 3, Mut68

To reveal the genetic mechanism behind improved growth and lipid accumulation, we determined the disrupted gene in Mut68 by performing whole genome sequencing. We found the sequence of the pNs*sh*ble plasmid, and it was integrated into the gene encoding TPS, named *Ns*TPS (Fig. 4a, Additional file 5: Fig. S2). *Ns*TPS is composed of two domains: the TPS domain in the N-terminal half and the TPP domain in the C-terminal half, where the latter was found to be disrupted by the mutagenic plasmid. The integration of this plasmid was confirmed by PCR using the following primers: F_TPP that binds in TPP and R_TPP that binds in pNs*Sh*ble (Additional file 1: Table S1). As expected, the 400-bp fragment of the PCR band was obtained only in Mut68, confirming the integration site in the TPP domain shown by whole genome sequencing (Fig. 4b). The TPP domain may have similar activity with trehalose-6-phosphate phosphatase (*otsB*) in bacteria, while the N-terminal TPS domain is predicted to function as trehalose-6-phosphate synthase (*otsA*) in bacteria [44]. It is thus expected that *Ns*TPS can carry out both functions, similar to other eukaryotic TPSs reported in yeast and plants [44].

### Protein structure analysis of WT and truncated TPS

To assess the actual consequences of disruption, we employed an *in silico* tool, named RaptorX, for the structural prediction of WT TPS and that of Mut68, as shown in Additional file 6: Fig. S3. The WT TPS protein was predicted to have four domains, comprising TPS_WT_ (H147–S634; *p* value: 9.02e^−11^), TPP_WT_ (K635–L917; *p*-value: 7.48e^−8^), N-terminal_WT_ (M1–A87; *p*-value: 4.51e^−3^), and C-terminal_WT_ (R1042–R1093; *p*-value: 1.76e^−3^). Among 1093 amino acids, 910 (83%) residues were modeled and 293 (26%) positions were predicted to be disordered using the protein database template 5HUT. TPS_WT_ was predicted to be alpha, alpha-trehalose-phosphate synthase (ααTPS; EC:2.4.1.15) and uridine-5′-diphosphate (UDP) and guanosine-5-diphosphate (GDP) were predicted to be potential ligands that can bind to the same pocket consisting of G176, V426, R428, K433, V461, V463, S504, I505, L510, M529, N530, L531, V532, and E535. TPP_WT_ was predicted to be trehalose-6-phosphate phosphatase (TPP; EC:3.1.3.12) and Mg^2+^ was predicted to be a ligand that binds to D659, Y660, D661, S699, G700, K821, and D854 (Additional file 7: Fig. S4). The function and ligand binding of N-terminal_WT_ and domain C-terminal_WT_ could not be predicted.

The truncated TPS protein in Mut68 was predicted to consist of TPS_Tr_ (K146–K635; *p*-value: 2.51e^−11^), TPP_Tr_ (L636–P799; *p*-value: 5.97e^−7^), and N-terminal_Tr_ (M1–A145; *p*-value: 4.93e^−3^) (Additional file 5: Fig. S2, Additional file 6: Fig. S3b). All of the 799 residues were modeled and 141 (17%) positions were predicted to be disordered, also using 5HUT as a template. TPS_Tr_ was predicted to have the same function and ligand-binding site with the TPS_WT_, although there was one residue difference. In contrast, TPP_Tr_ was predicted to have an unknown function, because it possesses only 58% of the residues of the TPP_WT_. Only four Mg^2+^ binding residues, D659, Y660, D661, G662, were predicted in TPP_Tr_, while there were three more Mg^2+^ binding residues in TPP_WT_. In addition, the protein function and ligand-binding pocket of N-terminal_Tr_ were predicted to be unknown.

### Expression of TPS and TPP domains of *Ns*TPS in Mut68

**Fig. 5:**
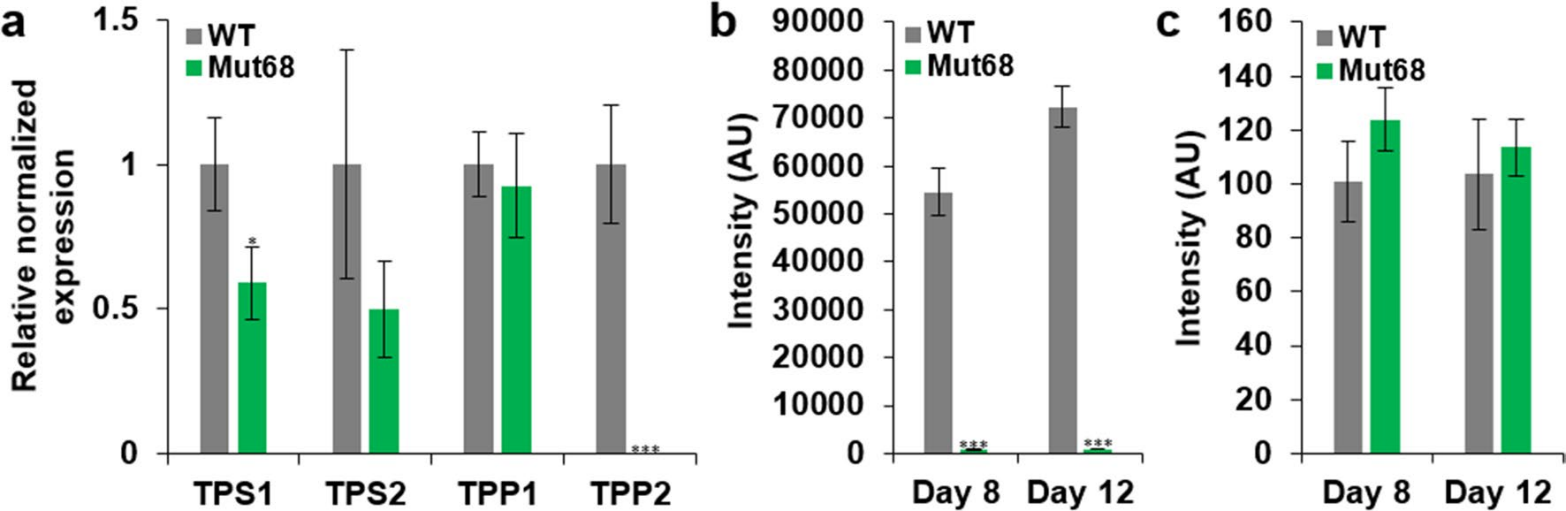
Expression of different area of TPS and TPP domains in *Ns*TPS, and metabolic consequences. **a** qRT-PCR was performed to show expression of TPS1 and TPS2 in the TPS domain, while TPP1 and TPP2 in the TPP domain. Metabolite profiling via GC–MS showed abundance of trehalose (**b**) and trehalose-6-phosphate (**c**) on days 8 and 12 in Mut68 compared to WT

We examined the expression of the TPS and TPP domains of *Ns*TPS by performing quantitative real-time PCR (qRT-PCR) with four sets of primers targeting different sites of the two domains (Fig. 5a). The TPS1 and TPS2 sites were located in the TPS domain, while the TPP1 and TPP2 sites were located in the TPP domain, as indicated in Fig. 4a. Among these sites, only TPP2 was downstream of the integration site of pNs*Sh*ble in Mut68. Even though these sites were located in the single gene of *Ns*TPS, only TPP2 expression was abolished in Mut68, while other sites were moderately reduced (Fig. 5a). This expression pattern suggested that the TPP function might have been lost in Mut68 while the TPS function was retained, which was further characterized by metabolomic analyses.

To reveal the differential functions of TPS and TPP domains, we performed metabolite profiling by employing GC–MS in Mut68 as shown in Fig. 5b, c. We focused on the quantitation of T6P and trehalose, since the TPP domain is involved in the formation of the intermediate metabolite T6P, while the TPS domain produces the final product trehalose. As predictable from the expression data, we found that the trehalose content was greatly reduced in Mut68 on days 8 and 12 of cultivation, probably because of the disruption of TPP. In contrast, the T6P content was rather increased moderately, albeit insignificantly, in Mut68, possibly by the incomplete conversion of T6P to trehalose. It is also possible that unknown metabolic and/or regulatory genes related to trehalose metabolism may have effects on the T6P level.

### Further metabolite profiling of amino acids

**Fig. 6:**
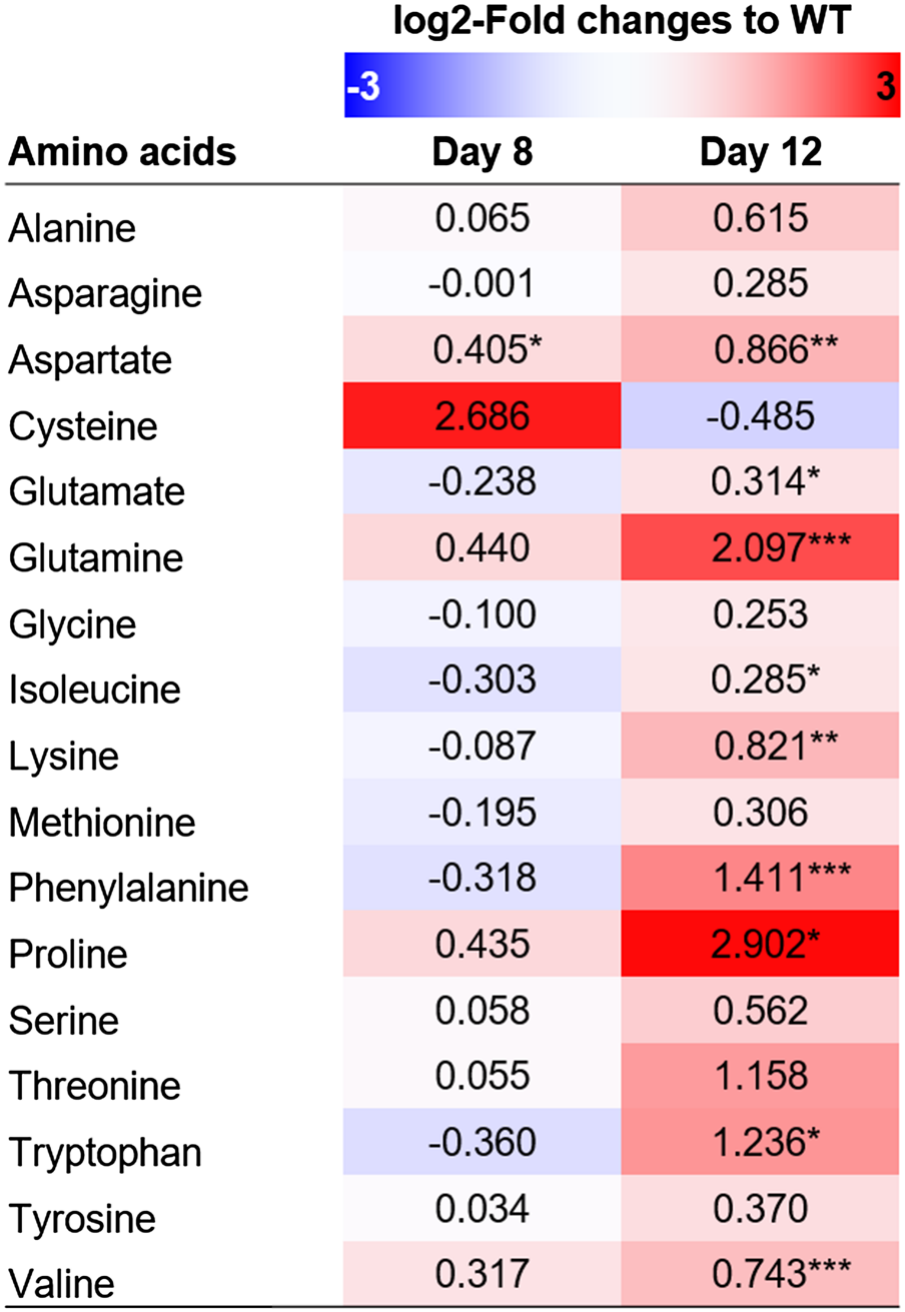
Metabolomic analyses of amino acids in Mut68

Trehalose is known to be involved in stress responses, particularly as an osmotic protectant and as a nutrient reserve in plants [45]. Mut68 contains greatly reduced trehalose contents, which can render it imbalanced in stress-related amino acids. In general, certain amino acids accumulate under stress conditions, as reported in plants [46], and certain stress conditions are known to induce lipid accumulation in microalgae [47]. Therefore, we also included amino acids in the metabolite profiling using GC–MS (Fig. 6) as described for trehalose analyses (Fig. 5b). Mut68 showed remarkable increase in certain amino acids: proline increased by 7.5-fold, glutamine by 4.3-fold, and tryptophan by 2.4-fold compared to WT. These increased amino acids were observed mainly on day 12, while no change was observed on day 8, except for cysteine, which showed an increase on day 8 only, even though no statistical significance was found. These data suggest that the greatly reduced trehalose content in Mut68 might have caused the accumulation of certain stress-related amino acids, which may be related to lipid accumulation.

### Phylogenetic analyses of *Ns*TPS with other TPSs from yeasts and plants

**Fig. 7:**
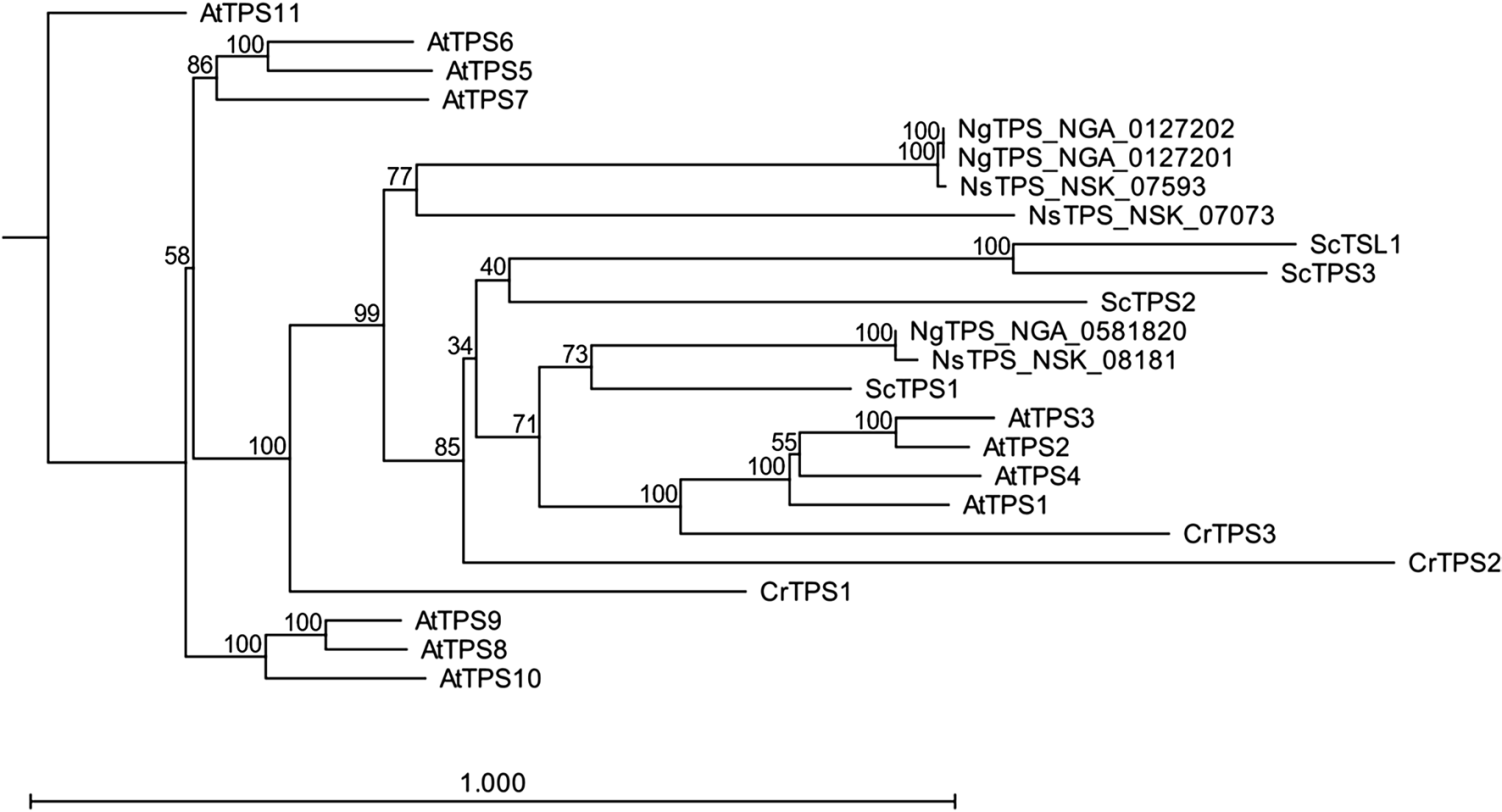
Phylogenetic tree of trehalose-6-phosphate synthase from *N. salina* (*Ns*TPS), *N. gaditana* (*Ng*TPS), *C*. *reinhardtii* (*Cr*TPS), *S. cerevisiae* (*Sc*TPS), and *A. thaliana* (*At*TPS)

Phylogenetic analysis was performed to further understand the functional aspects of *Ns*TPS compared to those of other known TPSs from other organisms, including *Nannochloropsis gaditana*, yeasts, and plants (Fig. 7). We obtained TPS-related sequences for *N. salina* from in-house genomic database, soon to be released in a comprehensive *Nannochloropsis* genomic resources (Jian Xu and colleagues, in preparation). These included three genes, *Ns*TPS_07073 (*Ns*TPS1), *Ns*TPS_07593 (*Ns*TPS2), and *Ns*TPS_08181, among which *Ns*TPS_08181 was disrupted in Mut68, which we named *Ns*TPS3. We also included other TPSs from *N. gaditana* (*Ng*TPS), *Arabidopsis thaliana* (*At*TPS), *C. reinhardtii* (*Cr*TPS), and *Saccharomyces cerevisiae* (*Sc*TPS), obtained from their respective databases or the NCBI nucleotide database. As shown in Fig. 5, *Ns*TPS3, which was the disrupted gene in Mut68, exhibited a close phylogenetic relationship with *Sc*TPS1 and *At*TPS1-4, which are known to produce trehalose [48]. This close relationship to known TPSs suggests that *Ns*TPS3 was actually involved in trehalose biosynthesis, which is consistent with our data. It should also be noted that *Ns*TPS3 would be a main *Ns*TPS for trehalose biosynthesis in *N. salina*, since Mut68 showed greatly reduced trehalose, which could not be complemented by other *Ns*TPSs.

## Discussion

In this study, we characterized Mut68 showing enhanced growth and lipid biosynthesis, isolated from insertional mutagenesis and FACS-based characterization. The insertional mutagenesis technique allowed the isolation of a novel gene that affected growth and lipid metabolism, in association with stress-related amino acids. Mut68 showed growth and lipid phenotypes comparable to those of previously engineered microalgal strains [15–17], suggesting that the insertional mutagenic technique is a valuable strategy to identify novel genes for strain development. We identified the gene responsible for Mut68 as TPS based on genomic sequencing, from which no other insertions were found, suggesting that only the TPS protein was disrupted in Mut68 by insertion of the pNs*Sh*ble. Consistent with this suggestion, we found only one integration based on Southern blot analyses (as shown in Fig. 2d). However, it is also possible that additional point mutations during transformation or standard lab practice might have accumulated and contributed to the phenotypes of Mut68 In this respect, this can be confirmed by complementation analyses using the WT copy of TPS in the future. Nonetheless, the TPS protein identified in our mutant screen might present a valuable target gene for the genetic engineering of microalgae and other organisms.

**Fig. 8:**
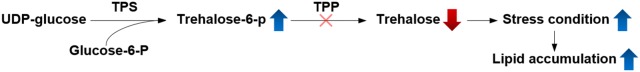
Trehalose metabolism-related lipid biosynthesis and growth enhancement

The TPP_WT_ domain plays a role as a TPP, which converts T6P and H_2_O into trehalose and phosphate [49]. According to Rao et al. Mg^2+^ plays an important role in the catalytic activity and/or structural integrity of TPP (Additional file 7: Fig. S4). However, four Mg^2+^ binding residues exist in the TPP_Tr_ domain of truncated TPS protein, while there are seven Mg^2+^ binding residues in the TPP_WT_ domain; thus, the ion-pair responsible for the catalytic activity might be unstable or absent. Thus, it seemed that truncated TPS only has ααTPS activity without TPP activity, probably leading to a loss of TPP function. The partial loss of function was consistent with our metabolomic analyses showing the loss of only trehalose (Fig. 5b).

TPS is required for the production of trehalose from UDP-glucose and glucose-6-phosphate, via T6P as an intermediate. The resulting trehalose has been known for its nutritional and protectant roles during and after stress in microorganisms and plants [50, 51]. It is also involved in stress alleviation in human diseases, including Parkinson’s and Huntington’s diseases [52]; however, its exact mechanism of function is not known. It is thought that trehalose can function as a “chemical chaperone” because of its ability to stabilize proteins and other molecular structures under stress conditions [53]. We found that Mut68 contained severely reduced amounts of trehalose because of the insertional disruption of *Ns*TPS3. For now, it is not clear how the lack of trehalose is associated with enhanced growth and lipid accumulation. This can be reconciled by our postulation that the lack of trehalose may mimic stress conditions leading to lipid accumulation, analogous to nitrogen stress-induced lipid accumulation (Fig. 8) [54]. However, Mut68 was clearly distinguishable from such simple stress conditions; growth was not compromised. Nonetheless, this beneficial phenotype is worth pursing to determine the mechanisms behind lipid accumulation without sacrificing growth.

It was also interesting to find the remarkable increase in certain amino acids in Mut68, mainly those of proline, glutamine, and tryptophan. Among these, proline is well known for its association with stress tolerance [46], which may have been induced by the lack of trehalose in Mut68. Tryptophan can also be involved in stress response as a precursor to hormones such as melatonin and auxin [55], which can be involved in stress tolerance as well as developmental regulation in animals and plants. Similarly, glutamine is known to be a precursor to proline biosynthesis [56], leading to its secondary involvement in stress responses. The mechanism behind the increased amino acid contents in Mut68 and their function is not clear, but it can be speculated that these amino acids might have contributed to improved growth, since they are also involved in normal cellular functions [56].

Mut68 also showed a moderate increase in T6P, the intermediate in trehalose biosynthesis. Interestingly, T6P has been reported to have functions distinct from trehalose, including the regulation of sucrose and other carbohydrate metabolites in plant cells [57]. It is known that T6P is involved in carbohydrate utilization in higher plant cells, such as those of *A. thaliana*, and increases cell growth rate through the regulation of glycolysis [57]. In addition, in WT plant cells, high sucrose levels have been reported to be associated with high trehalose-6-phosphate levels and promoted cell growth [58]. In addition, T6P positively regulates fatty acid synthesis and photosynthesis in *A. thaliana* [59, 60]. Therefore, the increase in T6P could also contribute to improved growth and lipid accumulation in Mut68.

Cell growth is generally related to photosynthetic efficiency, and we thus measured the maximum quantum yield of PSII and non-photochemical quenching using a pulse–amplitude modulation device [61]. However, we did not find any difference between WT and Mut68, suggesting that the enhanced growth in Mut68 was not caused by improved photosynthesis (data not shown).

Taken together, we have isolated an insertional mutant, Mut68, which showed improved growth and lipid production, with disruption at the *Ns*TPS3 locus. Supporting this improvement, Mut68 showed increased level of stress-related amino acids and T6P. This is a successful demonstration that insertional mutagenesis can be employed in the identification of new genes for the improved production of biomass and lipids in unexplored organisms, such as *Nannochloropsis* and other microalgae. It should also be noted that TPS can be considered a novel target for the genetic engineering of microalgae for increased lipid production without the compromise of decreased growth. This has become particularly important since gene editing technology, including CRISPR, has been made available for use with microalgae [22, 62, 63].

## Conclusions

In this study, we obtained a *N. salina* mutant, Mut68, which showed simultaneous increases in the specific growth rate by 53% and FAME productivity by 75%, isolated from an insertional mutagenesis library and characterized by FACS. Mut68 contained a single integration of the mutagenic plasmid at the TPP domain of *Ns*TPS3. Accordingly, metabolic analyses showed a 99% reduction in the intracellular level of trehalose, while showing a moderate increase in the intermediate T6P, together with increased levels of other stress-related amino acids. These chemical changes have not been reported in *Nannochloropsis*, which may have contributed to the improved lipid production without compromised growth. Conclusively, we present an excellent proof of concept that insertional mutagenesis can be employed in genetic screens for the identification of novel and unknown genes for improving traits in biomass and lipid production. We also conclude that TPS is a good target for gene editing technologies for the improvement of microalgae and possibly other organisms.

## Methods

### Microorganisms and culture condition

*Nannochloropsis salina* CCMP1776 (National Center for Marine Algae and Microbiota) was cultured in modified F2N medium which was composed of 15 g/L sea salt (Sigma-Aldrich, St. Louis, Mo, USA), 427.5 mg/L NaNO_3_, 30 mg/L NaH_2_PO_4_·2H_2_O, 5 mL/L trace metal mixture (4.36 g/L Na_2_ EDTA·H_2_O, 3.15 g/L FeCl_3_·6H_2_O, 10 mg/L CoCl_2_·6H_2_O, 22 mg/L ZnSO_4_·7H_2_O, 180 mg/L MnCl_2_·4H_2_O, 9.8 mg/L CuSO_4_·5H_2_O, and 6.3 mg/L Na_2_MoO_4_·2H_2_O), 10 mM Tris–HCl (pH 7.6), and 2.5 mL/L vitamin stock solution (1 mg/L vitamin B_12_, 1 mg/L biotin, and 200 mg/L thiamine·HCl) [64]. Microalgal cells were cultivated in 250-mL Erlenmeyer baffled flasks containing 200 mL modified F2N medium at 25 °C with shaking (120 rpm) under fluorescent light (120 μmol photons/m^2^/s). Air containing 2% (v/v) CO_2_ was supplied into the culture at 0.5 vvm (volume gas per volume medium per minute).

*Escherichia coli* XL1-blue (*recA1 endA1 gyrA96 thi*-*1 hsdR17 supE44 relA1 lac* [F’ *proAB lacI*^q^ ZΔ*M15* Tn*10* (Tet^r^)]) was used as a host for gene cloning. *E. coli* were cultured in Luria–Bertani (LB) medium (BD Difco, Franklin Lakes, NJ, USA) composed of 10 g/L tryptone, 5 g/L yeast extract, and 10 g/L NaCl supplemented with 100 μg/mL ampicillin sodium salt (Sigma-Aldrich) and kanamycin sulfate from *Streptomyces kanamyceticus* (Sigma-Aldrich) at 37 °C with shaking (200 rpm).

### Plasmid manipulation

The plasmid pNs*Sh*ble, containing the Zeocin-resistance marker gene flanked by the constitutive ubiquitin extension protein promoter and terminator, was constructed based on pNssfCherry [65]. The pNssfCherry vector was digested with the *Eco*RI restriction enzyme to remove the sequence of the constitutive β-tubulin (TUB) promoter, coding sequence of sfCherry, and TUB terminator. All restriction enzymes used in this study were purchased from Enzynomics (Deajeon, Korea). The digested plasmid was self-ligated and transformed into *E. coli* XL1-blue competent cells using a MicroPulser™ Electroporator (Bio-Rad, Hercules, CA, USA).

### Insertional mutagenesis

The insertional mutagenesis to construct the library was conducted by the electroporation of pNs*Sh*ble into *N. salina* [27, 66]. The pNs*Sh*ble plasmid was linearized by treating the *Nco*I restriction enzyme and 1 μg/μL linearized pNs*Sh*ble was prepared in concentration. Microalgal cells were cultured in modified F2N medium and harvested at the mid-exponential phase (OD_680_ = 6) [67]. Harvested cells were rinsed four times using 375 mM sorbitol (Sigma-Aldrich) by centrifugation (6000 rpm, 10 min at 4 °C). Washed cell pellets were resuspended in 375 mM sorbitol with a final cell concentration of 5 × 10^9^ cells/mL. Then, 50 μL of microalgal cells and 2.5 μg of linearized pNs*Sh*ble vector were added to 2 mm cuvettes (BTX, MA, USA), and then electroporation was conducted by using BTX ECM 830 Electro Square Porator™ (12,000 V/cm; pulse length, 100 μs; number of pulses, 50; field strength, 50 μF; capacitance, 500 Ω). After electroporation, the cells were resuspended in 10 mL of modified F2N medium and recovered overnight in a dark room at 25 °C without shaking. The recovered cells were harvested by centrifugation (4000 rpm, 15 min at 25 °C) and plated on a modified F2N agar medium containing 2.5 μg/mL Zeocin (Thermo Fisher Scientific, MA, USA). The plated cells were incubated under fluorescent light (120 μmol photons m^−2^ s^−1^) for 4 weeks and colonies were chosen for library screening.

### Screening of insertional mutagenesis library by using FACS

All of the microalgal colonies that appeared on the modified F2N medium were selected and the microalgal cells were inoculated into modified F2N medium. After 7 days, 1 × 10^6^ microalgal cells were stained with 1 μM BODIPY 505/515 (4,4-difluoro-1,3,5,7-tetramethyl-4-bora-3a,4a-diaza-s-indacen; Sigma-Aldrich) and 0.2% (v/v) dimethyl sulfoxide (Sigma-Aldrich) in a dark room at 25 °C for 30 min, as reported previously [68]. The fluorescence intensities of stained cells were analyzed using the MoFlo XDP cell sorter (Beckman Coulter, Fullerton, CA, US). A 488-nm argon laser was used to excite the stained cells and emission signals from the cells were observed in the FL1 channel centered at 530–540 nm. The values of mean fluorescence intensity were analyzed using SUMMIT software version 5.2.

### PCR analysis of mutant

Microalgal colonies on plate were harvested and crude genomic DNA was extracted from the cells by using Instagene Matrix (Bio-Rad). 200 μL of Instagene matrix was added to the harvested cell pellets and incubated at 56 °C heating block for 20 min. Then, samples were gently vortexed for 10 s and incubated at 100 °C hot plate for 8 min. After heating, samples were centrifuged, and crude genomic DNA was prepared from supernatant was used as a template for PCR. F_ *Sh*ble and R_ *Sh*ble oligonucleotides were used for amplification of *Sh*-bleoR gene integrated into *N. salina* transformants (Additional file 1: Table S1). To detect gene of 18S rDNA, SR6 and SR9 oligonucleotides were used as reported previously [15]. PrimeSTAR HS Polymerase (Takara Bio Inc., Shiga, Japan) was used for the PCR with a C1000™ Thermal Cycler (Bio-Rad).

### Southern blot analysis

Southern blot analysis was performed by using the DIG-High Prime DNA Labeling and Detection Starter Kit II (Roche, Basel, Switzerland) and the genomic DNA samples for Southern blotting were prepared by the protocol previously described [69]. The microalgal cells reaching the stationary phase were centrifuged and washed with 50 mM ethylenediaminetetraacetic acid (EDTA). After washing, the supernatants were discarded and 150 μL of distilled water was added to resuspend the microalgal cell pellets. Then,  μL of SDS-EB was added to tubes and vortexed. 500 μL of phenol and chloroform (1:1, v/v) mixture was added to samples, followed by vortexing for 5 min and centrifugation at 13,000 rpm for 5 min. After centrifugation, the supernatants were transferred into new tubes. The mixture of phenol and chloroform was added to the supernatant and then the samples were vortexed for 5 min, followed by centrifugation 13,000 rpm for 5 min. The supernatants obtained after centrifugation at 13,000 rpm for 5 min were transferred to new tubes, and 500 μL of chloroform was added thereto, followed by vortex for 5 min, followed by centrifugation at 13,000 rpm for 5 min. 400 μL of the supernatant was taken and moved to the new tubes and 800 μL of ethanol was added to the supernatant. The mixtures were incubated at room temperature for 15 min to precipitate genomic DNA. The obtained DNA pellet was rinsed with 70% ethanol, and the air-dried DNAs were dissolved in 40 μl of TE buffer. 10 μg of genomic DNAs and 100 ng of pNs*Sh*ble vector were digested by *Nhe*I and *Xho*I restriction enzymes and analyzed to 0.8% (w/v) agarose gels by electrophoresis. After electrophoresis, the gel was rinsed with 0.25 M HCl for acid hydrolysis, 0.5 M NaOH, 1.5 M NaCl for denaturation, 0.5 M Tris–HCl pH 8.0, 1.5 M NaCl for neutralization. The separated DNA samples in the gel were transferred to a Hybond-N^+^ nylon membrane (GE Healthcare Life Sciences, Amersham, UK) by performing capillary transfer in 10 × SSC (3.0 M NaCl, 0.3 M NaC_6_H_8_O_7_ pH 7.0) overnight at room temperature. After UV-cross linking, the nylon membrane with transferred DNAs was pre-hybridized by the DIG-Easy-Hyb solution for 30 min at 54 °C [65]. The *Sh*-bleoR gene-specific DNA probe was amplified by PCR using F_ *Sh*ble and R_ *Sh*ble oligonucleotides (Additional file 1: Table S1) from pNssfGFP as a template DNA and then the DIG-labeled *Sh*-bleoR probe was added to the DIG-Easy-Hyb solution for hybridization on the DNAs in nylon membrane at 54 °C overnight. After probe hybridization, nylon membrane was stringently washed with 0.5 × SSC with 0.1% (w/v) sodium dodecyl sulfate (SDS) buffer at 65 °C for 15 min. The membrane was incubated with anti-digoxigenin-alkaline phosphatase conjugate antibody (Roche). The chemiluminescence reagents were treated on the membrane and immunodetective bands were visualized using the Chemidoc imaging system (Bio-Rad).

### Fatty acid methyl ester (FAME) analysis

A modified Folch’s method was used for FAME analysis [15, 25]. The 10 mg of lyophilized microalgal cells were mixed with chloroform–methanol solvent mixture (2:1, v/v) and vortexed vigorously for 10 min. As an internal standard, 0.5 mg of heptadecanoic acid (C17:0) was added to vortexed samples and 1 mL of methanol and 300 μL of sulfuric acid were added to sample in order. The samples were incubated in hot plate for 20 min at 100 °C for transesterification. After cooling the samples in room temperature, 1 mL of deionized water was added to each of samples and samples were vortexed for 5 min. After centrifugation (4000 rpm for 10 min, at 25 °C), the separated lower layer for organic phase was taken and filtered by using a 0.20 μm RC-membrane syringe filter (Sartorius Stedim Biotech, Germany). FAMEs in organic phase were detected via gas chromatograph (GC) (HP 6890, Agilent, Wilmington, DE, USA) with an HP-INNOWax polyethylene glycol column (HP 19,091 N-213, Agilent) and a flame ionization detector (FID). The temperature of GC oven was increased from 50 to 250 at 15 °C per min. Composition and contents of FAME in the sample were determined by comparison with a 37-component mix of FAME standards (F.A.M.E. MIX C8-C24, Supelco, USA).

### Whole genome sequencing

According to the protocol previously provided [15, 62], next-generation sequencing was performed on microalgal genomic DNA by using Illumina HiSeq 2000 (Illumina, San Diego, CA, USA). A paired-end sequencing platform (Seeders, Korea) was used to read the genomic DNA sequences. The SolexaQA package software (version 1.13) was used to trim short reading sequences to improve the sequencing quality. The DynamicTrim module was used to remove the lower quality base. To verify the insertion of pNs*Sh*ble, PCR was performed using F_TPS and R_TPS (Additional file 1: Table S1).

### Protein structure analysis of WT and truncated TPS

Protein structure analysis of WT and truncated TPS were carried out based on template-based protein structure modeling tool, RaptorX [70]. The amino acid sequence of WT TPS and truncated TPS were submitted to RaptorX Structure Prediction function for the protein structure modeling and ligand-binding site analysis. Protein structures were modeled based on the 5HUT structure of the trehalose-6-phosphate synthase in complex with UDP-glucose isolated from *Candida albicans* [71]. Final figures were made in the program PyMOL v2.3 (The PyMOL Molecular Graphics System, Version 2.0 Schrödinger, LLC).

### Quantitative real-time PCR (qRT-PCR)

The transcription level of each domain of *Ns*TPS was analyzed by conducting qRT-PCR as previously described [15]. The WT and Mut68 cell pellets were harvested and RNAs were extracted from the cells using NucleoZOL reagent (Macherey–Nagel, Germany), according to the protocol provided by the manufacturer. The remaining DNA in the RNA samples was removed using DNA-free™ DNase kits (Ambion, Austin, TX, USA). The reverse transcription of RNA samples was conducted to prepare cDNA samples using Superscript™ III Reverse Transcriptase and an Oligo(dT)_20_ Primer (Invitrogen, Carlsbad, CA, USA). The oligonucleotides used in qRT-PCR are listed in Additional file 1: Table S1. Then, 20 ng of prepared cDNA, 10 μM oligonucleotides, and 10 μL of Universal SYBR Supermix (Bio-Rad) were used for qRT-PCR, which was performed in accordance with a previous report [16].

### Phylogenetic tree construction

The phylogenetic tree of trehalose-6-phosphate synthase was constructed according to a previous report [17]. Trehalose-6-phosphate synthase from *N. salina*, *N. gaditana*, C. *reinhardtii*, *S. cerevisiae*, and *A. thaliana* were aligned, and the phylogenetic tree was drawn using the maximum likelihood and neighbor-joining methods, provided in CLC Bio Main Workbench.

### Metabolite extraction

After cultivation, 70% methanol was added at a 1:1 volume ratio of cell culture for quenching. Cells were harvested (10 million per sample), lyophilized, and stored at − 80 °C before further analysis. The lyophilized cells were ground with a single steel ball (5 mm i.d.) using Mixer Mill MM400 (Retsch GmbH & Co., Germany) followed by the addition of extraction solvent (methanol:isopropanol:water, 3:3:2, v/v/v, 1500 μL). The mixture was sonicated (5 min) and centrifuged for 5 min (13,200 rpm at 4 °C). The supernatant (1400 μL) was collected and transferred to a new 1.5-mL tube. The aliquot was concentrated to complete dryness in a speed vacuum concentrator (SCANVAC, Korea) and stored at − 80 °C before derivatization and mass spectrometric analysis [72].

### Untargeted primary metabolite profiling

The dried extract was derivatized with 5 µL (40 mg/mL) of pyridine (Thermo Fisher Scientific) in methoxyamine hydrochloride (Sigma-Aldrich) (200 rpm, 90 min at 30 °C). Following the first derivatization, the derivative was mixed with 2 µL of FAMEs and 45 µL of N-methyl-N-trimethylsilyltrifluoroacetamide (MSTFA + 1% trimethylchlorosilane, Thermo Fisher Scientific), and incubated for 60 min at 37 °C (200 rpm) [73].

Gas-chromatographic separation and mass spectrometric analysis were conducted using an Agilent 7890B Gas Chromatograph and a LECO Pegasus HT time-of-flight mass spectrometer, according to our previous studies [72, 74]. Data were preprocessed upon data acquisition (e.g., entire spectrum, retention time, and purity) by ChromaTOF software (ver. 4.5) and then post-processed using the *BinBase* algorithm [75, 76]. The algorithm collected deconvoluted data, validated the spectra (unique ion and all apex masses), calculated the retention index based on FAMEs, and annotated the peaks against the Fiehn and NIST libraries.

## Supplementary information

**Additional file 1: Table S1.** Oligonucleotides used in this study.

**Additional file 2: Fig. S1.** Whole gel Southern blot result. Lane 1, WT (no digestion); lane 2, Mut68 (no digestion); Lane 3, WT (digested by *Nhe*I); lane 4, Mut68 (digested by *Nhe*I); Lane 5, WT (digested by *Nhe*I and *Xho*I); lane 6, Mut68 (digested by *Nhe*I and *Xho*I).

**Additional file 3: Table S2.** Dry cell weight and biomass productivity analysis of WT and Mut68.

**Additional file 4: Table S3.** Comparison of FAME composition of WT and Mut68.

**Additional file 5: Fig. S2.** Coding DNA sequence of truncated TPS in Mut68. Inserted pNs*Sh*ble was highlighted in green and stop codon generated by insertion of pNs*Sh*ble was underlined.

**Additional file 6: Fig. S3.** Predicted protein crystal structure of WT (**a**) and truncated (**b**) TPS based on the template-based prediction tool RaptorX. Yellow, TPS_WT_ and TPS _Tr_ domain; cyan, TPP_WT_ and TPP_Tr_ domain; purple, N-terminal_WT_ and N-terminal_Tr_ domain; orange, C-terminal_WT_; grey, disordered region.

**Additional file 7: Fig. S4.** Predicted binding pocket in WT TPP domain. The Mg^2+^ ion binding site of WTTPP domain was expressed.

## Data Availability

All data generated or analyzed during this study are included in this published article.

## Abbreviations

*Ns*TPS: Trehalose-6-phosphate synthase in *N. salina* containing TPS and TPP domain
TPS domain: Trehalose-6-phosphate synthase domain
TPP domain: Trehalose-6-phosphate phosphatase domain
FACS: Fluorescence-activated cell sorting
FAME: Fatty acid methyl ester

## References

[1] Congressional research service, the renewable fuel standard (RFS): an overview. 2019. (https://fas.org/sgp/crs/misc/R43325.pdf).

[2] Dang NM, Lee K (2018). Utilization of organic liquid fertilizer in microalgae cultivation for biodiesel production. Biotechnol Bioproc E..

[3] Chen F (1996). High cell density culture of microalgae in heterotrophic growth. Trends Biotechnol.

[4] Khan MI, Shin JH, Kim JD (2018). The promising future of microalgae: current status, challenges, and optimization of a sustainable and renewable industry for biofuels, feed, and other products. Microb Cell Fact.

[5] Ma Y, Wang Z, Yu C, Yin Y, Zhou G (2014). Evaluation of the potential of 9 *Nannochloropsis* strains for biodiesel production. Bioresour Technol.

[6] Festel G, Bellof M, Würmseher M, Rammer C, Boles E, Domingos Padula A, Silveira dos Santos M, Benedetti Santos OI, Borenstein D (2014). Calculation of raw material prices and conversion costs for biofuels. Liquid biofuels: emergence, development and prospects.

[7] Sun X-M, Ren L-J, Zhao Q-Y, Ji X-J, Huang H (2019). Enhancement of lipid accumulation in microalgae by metabolic engineering. Biochem Biophys Acta.

[8] Jeon S, Lim JM, Lee HG, Shin SE, Kang NK, Park YI, Oh HM, Jeong WJ, Jeong BR, Chang YK (2017). Current status and perspectives of genome editing technology for microalgae. Biotechnol Biofuels.

[9] Park S, Nguyen THT, Jin E (2019). Improving lipid production by strain development in microalgae: strategies, challenges and perspectives. Bioresour Technol.

[10] Rengel R, Smith RT, Haslam RP, Sayanova O, Vila M, León R (2018). Overexpression of acetyl-CoA synthetase (ACS) enhances the biosynthesis of neutral lipids and starch in the green microalga *Chlamydomonas reinhardtii*. Algal Res.

[11] Li D-W, Cen S-Y, Liu Y-H, Balamurugan S, Zheng X-Y, Alimujiang A, Yang W-D, Liu J-S, Li H-Y (2016). A type 2 diacylglycerol acyltransferase accelerates the triacylglycerol biosynthesis in heterokont oleaginous microalga *Nannochloropsis oceanica*. J Biotechnol.

[12] Wang X, Dong H-P, Wei W, Balamurugan S, Yang W-D, Liu J-S, Li H-Y (2018). Dual expression of plastidial GPAT1 and LPAT1 regulates triacylglycerol production and the fatty acid profile in *Phaeodactylum tricornutum*. Biotechnol Biofuels.

[13] Xue J, Balamurugan S, Li D-W, Liu Y-H, Zeng H, Wang L, Yang W-D, Liu J-S, Li H-Y (2017). Glucose-6-phosphate dehydrogenase as a target for highly efficient fatty acid biosynthesis in microalgae by enhancing NADPH supply. Metab Eng.

[14] Yoneda K, Yoshida M, Suzuki I, Watanabe MM (2018). Homologous expression of lipid droplet protein-enhanced neutral lipid accumulation in the marine diatom *Phaeodactylum tricornutum*. J Appl Phycol.

[15] Kang NK, Jeon S, Kwon S, Koh HG, Shin S-E, Lee B, Choi G-G, Yang J-W, Jeong B-R, Chang YK (2015). Effects of overexpression of a bHLH transcription factor on biomass and lipid production in *Nannochloropsis salina*. Biotechnol Biofuels..

[16] Kang NK, Kim EK, Kim YU, Lee B, Jeong W-J, Jeong B-R, Chang YK (2017). Increased lipid production by heterologous expression of AtWRI1 transcription factor in *Nannochloropsis salina*. Biotechnol Biofuels..

[17] Kwon S, Kang NK, Koh HG, Shin S-E, Lee B, Jeong B-R, Chang YK (2018). Enhancement of biomass and lipid productivity by overexpression of a bZIP transcription factor in *Nannochloropsis salina*. Biotechnol Bioeng..

[18] Bajhaiya AK, Dean AP, Zeef LAH, Webster RE, Pittman JK (2016). PSR1 is a global transcriptional regulator of phosphorus deficiency responses and carbon storage metabolism in *Chlamydomonas reinhardtii*. Plant Physiol.

[19] Daboussi F, Leduc S, Maréchal A, Dubois G, Guyot V, Perez-Michaut C, Amato A, Falciatore A, Juillerat A, Beurdeley M, Voytas DF, Cavarec L, Duchateau P (2014). Genome engineering empowers the diatom *Phaeodactylum tricornutum* for biotechnology. Nat Commun..

[20] Deng X, Cai J, Li Y, Fei XJBL (2014). Expression and knockdown of the PEPC1 gene affect carbon flux in the biosynthesis of triacylglycerols by the green alga *Chlamydomonas reinhardtii*. Biotechnol Lett.

[21] Shin YS, Jeong J, Nguyen THT, Kim JYH, Jin E, Sim SJ (2019). Targeted knockout of phospholipase A2 to increase lipid productivity in *Chlamydomonas reinhardtii* for biodiesel production. Bioresour Technol.

[22] Ajjawi I, Verruto J, Aqui M, Soriaga LB, Coppersmith J, Kwok K, Peach L, Orchard E, Kalb R, Xu W, Carlson TJ, Francis K, Konigsfeld K, Bartalis J, Schultz A, Lambert W, Schwartz AS, Brown R, Moellering ER (2017). Lipid production in *Nannochloropsis gaditana* is doubled by decreasing expression of a single transcriptional regulator. Nat Biotechnol..

[23] Fu W, Nelson DR, Mystikou A, Daakour S, Salehi-Ashtiani K (2019). Advances in microalgal research and engineering development. Curr Opin Biotechnol.

[24] Poliner E, Farre EM, Benning C (2018). Advanced genetic tools enable synthetic biology in the oleaginous microalgae *Nannochloropsis* sp. Plant Cell Rep.

[25] Velmurugan N, Sung M, Yim SS, Park MS, Yang JW, Jeong KJ (2014). Systematically programmed adaptive evolution reveals potential role of carbon and nitrogen pathways during lipid accumulation in *Chlamydomonas reinhardtii*. Biotechnol Biofuels.

[26] Xie B, Stessman D, Hart JH, Dong H, Wang Y, Wright DA, Nikolau BJ, Spalding MH, Halverson LJ (2014). High-throughput fluorescence-activated cell sorting for lipid hyperaccumulating *Chlamydomonas reinhardtii* mutants. Plant Biotechnol J.

[27] Terashima M, Freeman ES, Jinkerson RE, Jonikas MC (2015). A fluorescence-activated cell sorting-based strategy for rapid isolation of high-lipid *Chlamydomonas* mutants. Plant J..

[28] Srinivas R, Ochs C (2012). Effect of UV-A irradiance on lipid accumulation in *Nannochloropsis oculata*. Photochem Photobiol.

[29] Beacham TA, Macia VM, Rooks P, White DA, Ali ST (2015). Altered lipid accumulation in *Nannochloropsis salina* CCAP849/3 following EMS and UV induced mutagenesis. Biotechnol Rep..

[30] de Jaeger L, Verbeek REM, Draaisma RB, Martens DE, Springer J, Eggink G, Wijffels RH (2014). Superior triacylglycerol (TAG) accumulation in starchless mutants of *Scenedesmus obliquus*: (I) mutant generation and characterization. Biotechnol Biofuels.

[31] Takeshita T, Ivanov IN, Oshima K, Ishii K, Kawamoto H, Ota S, Yamazaki T, Hirata A, Kazama Y, Abe T, Hattori M, Bisova K, Zachleder V, Kawano S (2018). Comparison of lipid productivity of *Parachlorella kessleri* heavy-ion beam irradiation mutant PK4 in laboratory and 150-L mass bioreactor, identification and characterization of its genetic variation. Algal Res.

[32] Kawaroe M, Sudrajat A, Hwangbo J, Augustine D (2015). Chemical mutagenesis of microalgae *Nannochloropsis* sp. using EMS (Ethyl Methanesulfonate). Br J Appl Sci Technol..

[33] Zhang Y, He M, Zou S, Fei C, Yan Y, Zheng S, Rajper AA, Wang C (2016). Breeding of high biomass and lipid producing *Desmodesmus* sp. by Ethylmethane sulfonate-induced mutation. Bioresour Technol..

[34] Doan TTY, Obbard JP (2012). Enhanced intracellular lipid in *Nannochloropsis* sp. via random mutagenesis and flow cytometric cell sorting. Algal Res..

[35] Perin G, Bellan A, Segalla A, Meneghesso A, Alboresi A, Morosinotto TJ (2015). Generation of random mutants to improve light-use efficiency of *Nannochloropsis gaditana* cultures for biofuel production. Biotechnol Biofuels.

[36] Kim JYH, Kwak HS, Sung YJ, Choi HI, Hong ME, Lim HS, Lee J-H, Lee SY, Sim SJ (2016). Microfluidic high-throughput selection of microalgal strains with superior photosynthetic productivity using competitive phototaxis. Sci Rep..

[37] Liu YG, Chen Y (2007). High-efficiency thermal asymmetric interlaced PCR for amplification of unknown flanking sequences. BioTechniques..

[38] Shin S-E, Koh HG, Kang NK, Suh WI, Jeong B-R, Lee B, Chang YK (2017). Isolation, phenotypic characterization and genome wide analysis of a *Chlamydomonas reinhardtii* strain naturally modified under laboratory conditions: towards enhanced microalgal biomass and lipid production for biofuels. Biotechnol Biofuels..

[39] González-Ballester D, de Montaigu A, Galván A, Fernández E (2005). Restriction enzyme site-directed amplification PCR: a tool to identify regions flanking a marker DNA. Anal Biochem.

[40] Mattanovich D, Borth N (2006). Applications of cell sorting in biotechnology. Microb Cell Fact.

[41] Yen Doan T-T, Obbard JP (2011). Enhanced lipid production in *Nannochloropsis* sp. using fluorescence-activated cell sorting. GCB Bioenergy..

[42] Brennan L, Blanco Fernandez A, Mostaert AS, Owende P (2012). Enhancement of BODIPY505/515 lipid fluorescence method for applications in biofuel-directed microalgae production. J Microbiol Methods.

[43] Velmurugan N, Sung M, Yim SS, Park MS, Yang JW, Jeong KJ (2013). Evaluation of intracellular lipid bodies in *Chlamydomonas reinhardtii* strains by flow cytometry. Bioresour Technol.

[44] Vandesteene L, Ramon M, Le Roy K, Van Dijck P, Rolland F (2010). A single active trehalose-6-P synthase (TPS) and a family of putative regulatory TPS-Like proteins in *Arabidopsis*. Mol Plant..

[45] John R, Raja V, Wani M, Jan N, Majeed U, Ahmad S, Yaqoob U, Kaul T (2017). Trehalose: Metabolism and Role in Stress Signaling in Plants. Stress Signaling Plants Genom Proteom Perspect..

[46] Batista-Silva W, Heinemann B, Rugen N, Nunes-Nesi A, Araújo WL, Braun H-P, Hildebrandt TM (2019). The role of amino acid metabolism during abiotic stress release. Plant, Cell Environ.

[47] Minhas AK, Hodgson P, Barrow CJ, Adholeya A (2016). A review on the assessment of stress conditions for simultaneous production of microalgal lipids and carotenoids. Front microbiol..

[48] Leyman B, Van Dijck P, Thevelein JM (2001). An unexpected plethora of trehalose biosynthesis genes in *Arabidopsis thaliana*. Trends Plant Sci.

[49] Rao KN, Kumaran D, Seetharaman J, Bonanno JB, Burley SK, Swaminathan S (2006). Crystal structure of trehalose-6-phosphate phosphatase–related protein: biochemical and biological implications. Protein Sci.

[50] Mahmud SA, Hirasawa T, Furusawa C, Yoshikawa K, Shimizu H (2012). Understanding the mechanism of heat stress tolerance caused by high trehalose accumulation in *Saccharomyces cerevisiae* using DNA microarray. J Biosci Bioeng.

[51] Chen Q, Haddad GG (2004). Role of trehalose phosphate synthase and trehalose during hypoxia: from flies to mammals. J Exp Biol.

[52] Lee HJ, Yoon YS, Lee SJ (2018). Mechanism of neuroprotection by trehalose: controversy surrounding autophagy induction. Cell Death Dis..

[53] Barati B, Gan S-Y, Lim P-E, Beardall J, Phang S-M (2019). Green algal molecular responses to temperature stress. Acta Physiol Plant.

[54] Magalhães RSS, Popova B, Braus GH, Outeiro TF, Eleutherio ECA (2018). The trehalose protective mechanism during thermal stress in *Saccharomyces cerevisiae*: the roles of Ath1 and Agt1. FEMS Yeast Res..

[55] Li J, Liu J, Zhu T, Zhao C, Li L, Chen M (2019). The role of melatonin in salt stress responses. Int J Mol Sci.

[56] Phang JM, Liu W, Hancock CN, Fischer JW (2015). Proline metabolism and cancer: emerging links to glutamine and collagen. Curr Opin Clin Nutr Metab Care..

[57] Figueroa CM, Lunn JE (2016). A tale of two sugars: trehalose 6-phosphate and sucrose. Plant Physiol.

[58] Schluepmann H, Berke L, Sanchez-Perez GF (2011). Metabolism control over growth: a case for trehalose-6-phosphate in plants. J Exp Bot.

[59] Zhai Z, Keereetaweep J, Liu H, Feil R, Lunn JE, Shanklin J (2018). Trehalose 6-phosphate positively regulates fatty acid synthesis by stabilizing Wrinkled1. Plant Cell..

[60] Oszvald M, Primavesi LF, Griffiths CA, Cohn J, Basu SS, Nuccio ML, Paul MJ (2018). Trehalose 6-phosphate regulates photosynthesis and assimilate partitioning in reproductive tissue. Plant Physiol.

[61] Koh HG, Kang NK, Jeon S, Shin SE, Jeong BR, Chang YK (2019). Heterologous synthesis of chlorophyll b in *Nannochloropsis salina* enhances growth and lipid production by increasing photosynthetic efficiency. Biotechnol Biofuels.

[62] Shin SE, Lim JM, Koh HG, Kim EK, Kang NK, Jeon S, Kwon S, Shin WS, Lee B, Hwangbo K, Kim J, Ye SH, Yun JY, Seo H, Oh HM, Kim KM, Kim JS, Jeong WJ, Chang YK, Jeong BR (2016). CRISPR/Cas9-induced knockout and knock-in mutations in *Chlamydomonas reinhardtii*. Sci Rep..

[63] Wang Q, Lu Y, Xin Y, Wei L, Huang S, Xu J (2016). Genome editing of model oleaginous microalgae *Nannochloropsis* spp. by CRISPR/Cas9. Plant J..

[64] Kilian O, Benemann CSE, Niyogi KK, Vick B (2011). High-efficiency homologous recombination in the oil-producing alga *Nannochloropsis* sp.. Proc Natl Acad Sci U S A..

[65] Kang NK, Choi GG, Kim EK, Shin SE, Jeon S, Park MS, Jeong KJ, Jeong BR, Chang YK, Yang JW, Lee B (2015). Heterologous overexpression of sfCherry fluorescent protein in *Nannochloropsis salina*. Biotechnol Rep (Amst)..

[66] Jeon S, Kang NK, Suh WI, Koh HG, Lee B, Chang YK (2019). Optimization of electroporation-based multiple pulses and further improvement of transformation efficiency using bacterial conditioned medium for *Nannochloropsis salina*. J Appl Phycol.

[67] Geis SW, Fleming KL, Korthals ET, Searle G, Reynolds L, Karner DA (2000). Modifications to the algal growth inhibition test for used a regulatory assay. Environ Toxicol Chem.

[68] Rumin J, Bonnefond H, Saint-Jean B, Rouxel C, Sciandra A, Bernard O, Cadoret JP, Bougaran G (2015). The use of fluorescent Nile red and BODIPY for lipid measurement in microalgae. Biotechnol Biofuels.

[69] Jeong B-R, Wu-Scharf D, Zhang C, Cerutti H (2002). Suppressors of transcriptional transgenic silencing in *Chlamydomonas* are sensitive to DNA-damaging agents and reactivate transposable elements. Proc Natl Acad Sci U S A..

[70] Xu J (2019). Distance-based protein folding powered by deep learning. Proc Natl Acad Sci U S A..

[71] Miao Y, Tenor JL, Toffaletti DL, Maskarinec SA, Liu J, Lee RE, Perfect JR, Brennan RG (2017). Structural and in vivo studies on trehalose-6-phosphate synthase from pathogenic fungi provide insights into its catalytic mechanism, biological necessity, and potential for novel antifungal drug design. mBio..

[72] Lee J-E, Cho YU, Kim KH, Lee DY (2016). Distinctive metabolomic responses of *Chlamydomonas reinhardtii* to the chemical elicitation by methyl jasmonate and salicylic acid. Process Biochem.

[73] Jang C-H, Lee G, Park Y-C, Kim KH, Lee DY (2017). Highly time-resolved metabolic reprogramming toward differential levels of phosphate in *Chlamydomonas reinhardtii*. J Microbiol Biotechnol.

[74] Fiehn O (2013). Metabolomic response of *Chlamydomonas reinhardtii* to the inhibition of target of rapamycin (TOR) by rapamycin. J Microbiol Biotechnol.

[75] Skogerson K, Wohlgemuth G, Barupal DK, Fiehn O (2011). The volatile compound BinBase mass spectral database. BMC Bioinformatics.

[76] Lee DY, Fiehn O (2008). High quality metabolomic data for *Chlamydomonas reinhardtii*. Plant methods..

