# Supplementary material for: Development and characterization of a Nannochloropsis mutant with simultaneously enhanced growth and lipid production
Source: Biotechnol Biofuels. 2020 Mar 5;13:38. doi: 10.1186/s13068-020-01681-4 (PMC7057510; doi:10.1186/s13068-020-01681-4)
Supplement: Supplementary file 1 — Additional file 1: Table S1. Oligonucleotides used in this study. [file 13068_2020_1681_MOESM1_ESM.docx]

**Table S1** Oligonucleotides used in this study

| Oligonucleotides | Sequence (5’→3’) |
| --- | --- |
| F_ *Sh*ble | AAGTTGACCAGTGCCGTTCCGGTG |
| R_ *Sh*ble | CTCGGTCACGAAGTGCACGCAGTT |
| SR6 | GTCAGAGGTGAAATTCTTGG |
| SR9 | AACTAAGAACGGCATGCAC |
| R-AT3 | GTGTTTCCCTCCATCGTG |
| R-AT4 | CCAGTTCGTCACAATACCG |
| F_TPP | AGCGAAACATCGCATCGAG |
| R_TPP | TTCCACCACCATCCCCTT |
| F_TPS_domain_1 | TGGGTCGAGTCCGACTACAT |
| R_TPS_domain_1 | TAGGTGTGAAAGCCGAGCAG |
| F_TPS_domain_2 | TATTCCTGACGCGACATCCG |
| R_TPS_domain_2 | TGCGTGATGCTCTGGTTGAT |
| F_TPP_domain_1 | GGTACCCTCACTCAGCTCCA |
| R_TPP_domain_1 | ATCTTCAGCTTGCCCAGCC |
| F_TPP_domain_2 | CGAGTACTTTACGGAGCGCA |
| R_TPP_domain_2 | CACCTCCACCATCCGATTCC |
