# Supplementary material for: Development and characterization of a Nannochloropsis mutant with simultaneously enhanced growth and lipid production
Source: Biotechnol Biofuels. 2020 Mar 5;13:38. doi: 10.1186/s13068-020-01681-4 (PMC7057510; doi:10.1186/s13068-020-01681-4)
Supplement: Supplementary file 2 — Additional file 2: Fig. S1. Whole gel Southern blot result. Lane 1, WT (no digestion); lane 2, Mut68 (no digestion); Lane 3, WT (digested by NheI); lane 4, Mut68 (digested by NheI); Lane 5, WT (digested by NheI and XhoI); lane 6, Mut68 (digested by NheI and XhoI). [file 13068_2020_1681_MOESM2_ESM.docx]

**
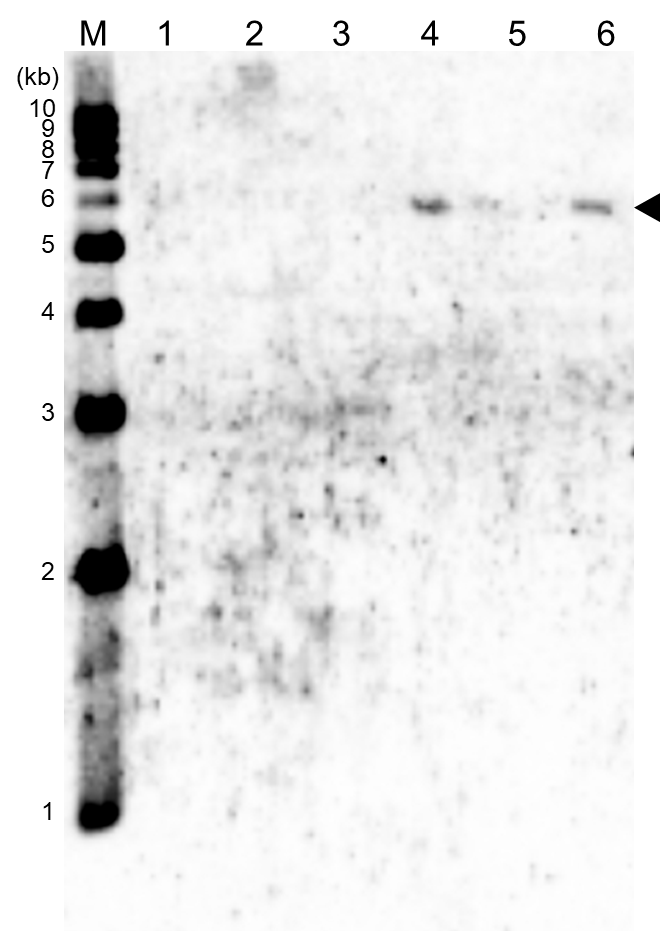
**

**Fig. S1** Whole gel southern blot result. Lane 1, WT (no digestion); lane 2, Mut68 (no digestion); Lane 3, WT (digested by *Nhe*I); lane 4, Mut68 (digested by *Nhe*I); Lane 5, WT (digested by *Nhe*I and *Xho*I); lane 6, Mut68 (digested by *Nhe*I and *Xho*I).
