# Supplementary material for: Development and characterization of a Nannochloropsis mutant with simultaneously enhanced growth and lipid production
Source: Biotechnol Biofuels. 2020 Mar 5;13:38. doi: 10.1186/s13068-020-01681-4 (PMC7057510; doi:10.1186/s13068-020-01681-4)
Supplement: Supplementary file 3 — Additional file 3: Table S2. Dry cell weight and biomass productivity analysis of WT and Mut68. [file 13068_2020_1681_MOESM3_ESM.docx]

**Table S2** Dry cell weight and biomass productivity analysis of wild-type *N. salina* and Mut68.

| Strain | Dry cell weight (g/L) | | Biomass productivity (mg/L/day) | |
| --- | --- | --- | --- | --- |
|  | Day 8 | Day 12 | Day 8 | Day 12 |
| WT | 1.20$\pm$0.10 | 2.30$\pm$0.14 | 150.0$\pm$12.3 | 191.7$\pm$11.34 |
| Mut68 | 1.56$\pm$0.17* | 2.83$\pm$0.50* | 194.8$\pm$21.1* | 235.4$\pm$42.0* |

The data shows the mean value of 4 samples. As determined by Student’s t-test, significant differences are indicated by asterisks (* P < 0.05, ** P < 0.01, *** P < 0.001).
