# Supplementary material for: Development and characterization of a Nannochloropsis mutant with simultaneously enhanced growth and lipid production
Source: Biotechnol Biofuels. 2020 Mar 5;13:38. doi: 10.1186/s13068-020-01681-4 (PMC7057510; doi:10.1186/s13068-020-01681-4)
Supplement: Supplementary file 4 — Additional file 4: Table S3. Comparison of FAME composition of WT and Mut68. [file 13068_2020_1681_MOESM4_ESM.docx]

**Table S3** Comparison of FAME composition of wild-type *N. salina* and Mut68.

|  | Day 8 |  | Day 12 |  |
| --- | --- | --- | --- | --- |
|  | WT | Mut68 | WT | Mut68 |
| C14:0 | 9.13$\pm$0.74 | 5.83$\pm$0.69*** | 4.56$\pm$1.14 | 2.78$\pm$0.32* |
| C16:0 | 25.56$\pm$2.47 | 29.72$\pm$1.17* | 38.36$\pm4.87$ | 40.10$\pm$0.66 |
| C16:1 | 27.16$\pm$1.00 | 31.21$\pm$0.44*** | 31.73$\pm$1.07 | 33.00$\pm$0.62* |
| C18:1 | 5.28$\pm$1.63 | 6.22$\pm$0.31 | 8.07$\pm$0.98 | 9.96$\pm$0.40* |
| C20:4n6 | 4.85$\pm$0.30 | 4.67$\pm$0.21 | 3.44$\pm$0.44 | 3.23$\pm$0.45 |
| C20:5 | 23.86$\pm$3.45 | 17.27$\pm$1.97* | 9.63$\pm$4.75 | 6.53$\pm$0.99 |
| C others | 4.07$\pm$1.53 | 4.46$\pm$2.24 | 4.10$\pm$0.67 | 3.65$\pm$1.11 |

The data shows the mean value of 4 samples. As determined by Student’s t-test, significant differences are indicated by asterisks (* P < 0.05, ** P < 0.01, *** P < 0.001).
