# Supplementary material for: Development and characterization of a Nannochloropsis mutant with simultaneously enhanced growth and lipid production
Source: Biotechnol Biofuels. 2020 Mar 5;13:38. doi: 10.1186/s13068-020-01681-4 (PMC7057510; doi:10.1186/s13068-020-01681-4)
Supplement: Supplementary file 5 — Additional file 5: Fig. S2. Coding DNA sequence of truncated TPS in Mut68. Inserted pNsShble was highlighted in green and stop codon generated by insertion of pNsShble was underlined. [file 13068_2020_1681_MOESM5_ESM.docx]

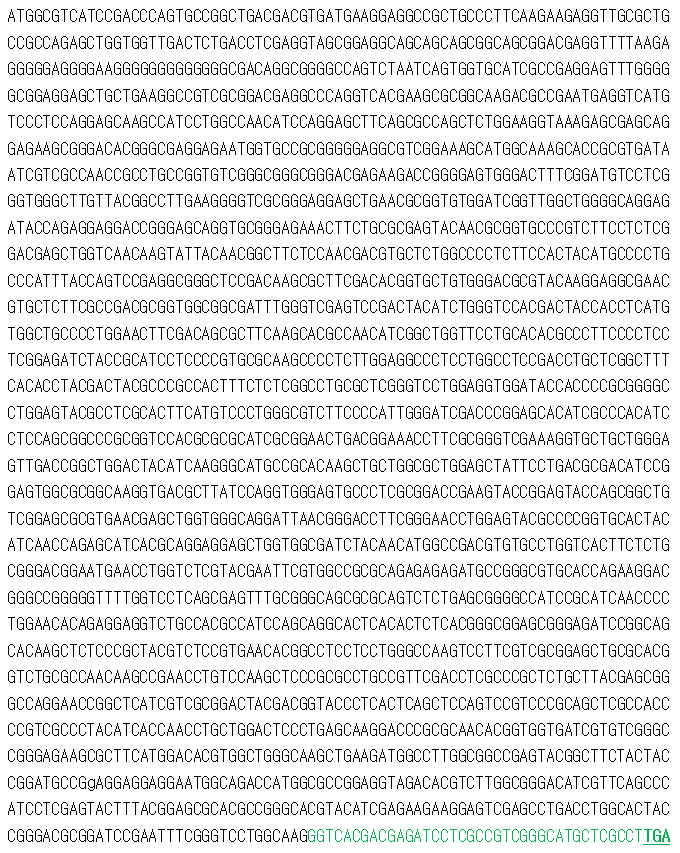
**Fig. S2** Coding DNA sequence of truncated TPS in Mut68. Inserted pNs*Sh*ble was highlighted in green and stop codon generated by insertion of pNs*Sh*ble was underlined.
