# Supplementary material for: Development and characterization of a Nannochloropsis mutant with simultaneously enhanced growth and lipid production
Source: Biotechnol Biofuels. 2020 Mar 5;13:38. doi: 10.1186/s13068-020-01681-4 (PMC7057510; doi:10.1186/s13068-020-01681-4)
Supplement: Supplementary file 6 — Additional file 6: Fig. S3. Predicted protein crystal structure of WT (a) and truncated (b) TPS based on the template-based prediction tool RaptorX. Yellow, TPSWT and TPS Tr domain; cyan, TPPWT and TPPTr domain; purple, N-terminalWT and N-terminalTr domain; orange, C-terminalWT; grey, disordered region. [file 13068_2020_1681_MOESM6_ESM.docx]

**
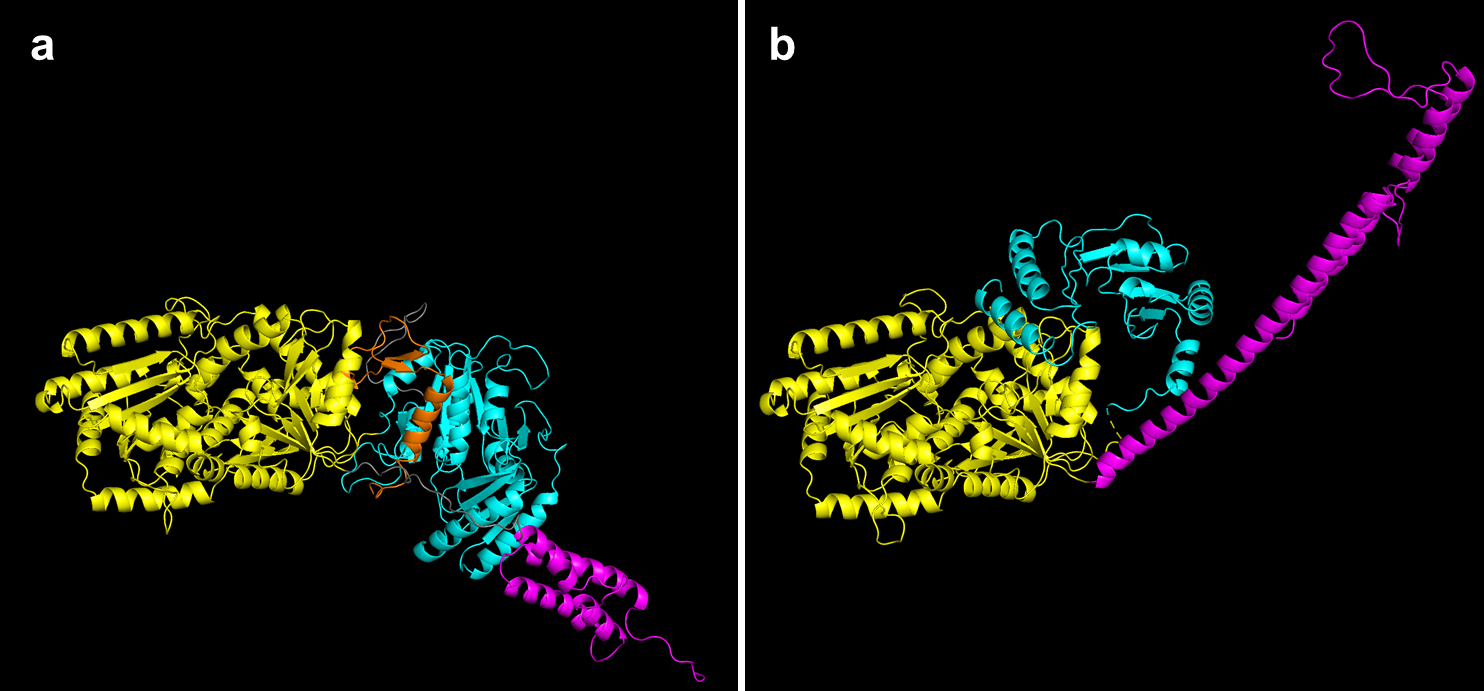
**

**Fig. S3** Predicted protein crystal structure of wild-type (**a**) and truncated (**b**) TPS based on the template-based prediction tool RaptorX. Yellow, TPS_WT_ and TPS _Tr_ domain; cyan, TPP_WT_ and TPP_Tr_ domain; purple, N-terminal_WT_ and N-terminal_Tr_ domain; orange, C-terminal_WT_; grey, disordered region.
