# Supplementary material for: Development and characterization of a Nannochloropsis mutant with simultaneously enhanced growth and lipid production
Source: Biotechnol Biofuels. 2020 Mar 5;13:38. doi: 10.1186/s13068-020-01681-4 (PMC7057510; doi:10.1186/s13068-020-01681-4)
Supplement: Supplementary file 7 — Additional file 7: Fig. S4. Predicted binding pocket in WT TPP domain. The Mg2+ ion binding site of WTTPP domain was expressed. [file 13068_2020_1681_MOESM7_ESM.docx]

**
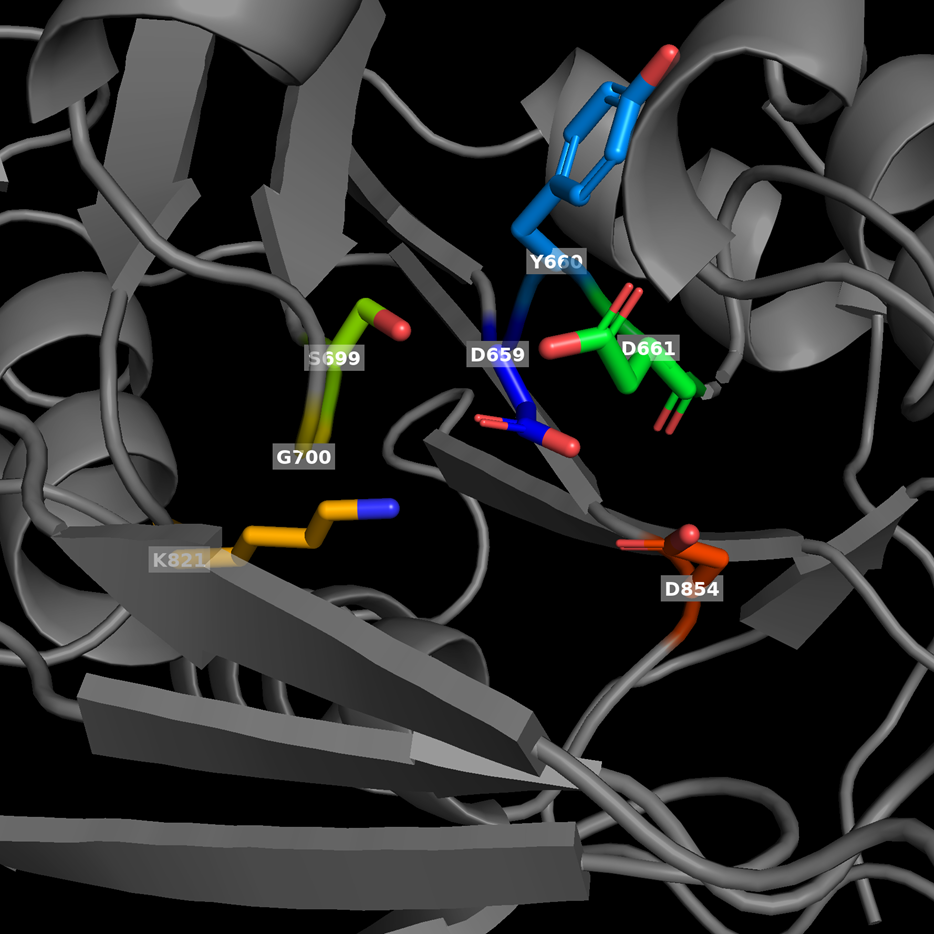
**

**Fig. S4** Predicted binding pocket in wild-type TPP domain. The Mg^2+^ ion binding site of wild type TPS was expressed.
